# Unilateral Mydriasis After Mandibular Fracture Fixation Surgery

**DOI:** 10.5812/aapm.13831

**Published:** 2014-03-16

**Authors:** Sholeh Nesioonpour, Kazem Khiabani, Marzieh Hassanijirdehi

**Affiliations:** 1 Department of Anesthesiology, Ahvaz Jundishapur University of Medical Sciences, Ahvaz, Iran; 2 Department of Oral and Maxillofacial Surgery, Ahvaz Jundishapur University of Medical Sciences, Ahvaz, Iran; 3 Department of Internal Medicine, Tehran University of Medical Sciences, Tehran, Iran

**Keywords:** Unilateral Mydriasis, Mandibular Fracture, Maxillofacial Surgery

## Abstract

**Introduction:**

Unilateral mydriasis is a seriously significant finding in neurologic examinations indicating life-threatening conditions such as cerebral vascular injuries.

**Case Presentation:**

A 24 year old woman with mandibular trauma was referred to our center after five days for a reduction of the right mandibular angle fracture. The patient had no history of any loss of consciousness after the accident. Her physical examination showed no abnormalities, except those related to her mandibular fracture. The laboratory results were normal as well. At 8:30 am a general anesthesia was induced. The patient’s eyes were kept shut throughout the surgical procedure. The operation included an intraoral open reduction and fixation using two miniplates without any complications. After the operation, it was noticed that the left eye was completely dilated with no reaction to light, while the right eye was normal. The management and outcomes in this patient were described in the present case report.

**Conclusions:**

Evaluating the size of the patient’s pupils before, during and after the operation, careful history, consult, CT scan and MRI would help to diagnosis. Although no probable cause was found to explain the transient mydriasis in our patient.

## 1. Introduction

Unilateral mydriasis is a seriously significant finding in neurologic examinations indicating life-threatening conditions such as cerebral vascular injuries, neoplastic masses, cerebral lesions or ophthalmologic injuries (1); however, in several case reports, this finding has been observed following application of some local anesthetic agents such as phenylephrine/lidocaine nasal spray (2), nasal cocaine/epinephrine application (3) or α-adrenergic drugs (4). Unilateral mydriasis results from an imbalance between the sympathetic and parasympathetic innervation of the eye, can be induced as a result of systemic causes (e.g. neurological and vascular disorders leading to increased intracranial pressure or following traumatic or hypoxic injuries to the autonomous system) and local causes (synechiae, congenital disorders of iris and pharmacological effects) (1). Furthermore, any drug able to impair the autonomous function of the eye can cause unilateral mydriasis; for example, in a study by D’Souza et al. epinephrine was the most likely causative agent which was injected into the nasal submucosa (5). In another study by Polomsky, application of a hemorrhoidal ointment to eyelids that contained an active ingredient known to produce mydriasis was the probable cause of unilateral mydriasis (6). In this case report, a new onset of unilateral mydriasis after mandibular fracture fixation surgery was presented.

## 2. Case Presentation

The patient was a 24-year-old female weighing 70 kg, with mandibular trauma as a result of a car accident without any loss of consciousness after the accident. Five days after the accident, the patient decided to undergo an open reduction and fixation surgery for the right mandibular angle fracture at Imam Khomeini hospital, Ahvaz, Iran. She had no history of any previous diseases except a history of an abortion under general anesthesia 6 years ago performed without any complications. The patient’s physical examination had no abnormal findings except paresthesia of the inferior lip, tenderness and edema in her right mandibular angle, forehead and scalp tenderness on the right side with no evident fracture. All nodes in the maxillofacial area except for the mandible itself were normal. There were no occlusion and the maximum jaw opening was 10 mm. The patient had a class II airway according to the Mallampati classification. As per the regulations of the maxillofacial ward, any patient with car accident should undergo a pre-operational neurosurgical consultation. Permission for performing the surgical procedure under general anesthesia was obtained from the patientduring the neurosurgical consultation. The laboratory analysis data were all in the normal acceptable ranges.

At 8:30 am, a general anesthesia was induced using 2 mg midazolam, 150 µg fentanyl, 100 mg lidocaine, 0.5 mg atropine, 300 mg thiopental sodium and 100 mg succinylcholine. Then the patient was intubated using a size 7.5 nasotracheal tube and 40 mg atracurium was administered for muscular relaxation. Tetracycline 1% ophthalmic ointment (Sina Darou, Tehran, Iran) was applied to the eyes, to keep the eyes shut during the operation. Anesthesia was maintained by infusion of 100-200 µg/kg/min propofol, 0.1 µg/kg/min remifentanil and 50% N_2_O- 50% O_2_. The patient underwent mechanical respiration and the monitoring included an electrocardiogram, noninvasive check of blood pressure and pulse oximetry implicated during the anesthesia. During the operation, the patient received 2000 mL of ringer's serum while the volume of bleeding was 100 mL and the patient released 150 mL urine. To maintain hemostasis, the surgeon locally injected 5 mL of 2% lidocaine with epinephrine 1:100000 after aspiration, into the right mandibular angle. After intraoral vestibular incision in mandibular angle area, displaced angle fracture was reduced and rigid fixation was performed using 2 four-hole miniplates at the external oblique ridge bythe Champy technique. After irrigation suturing with absorbable material was performed, the surgery and anesthesia were performed without any complications. At the end of the operation,the left pupil was completely dilated without any reaction to light while the right pupil was 3 mm with normal reaction to light. To reverse the muscular relaxation, 3.5 mg neostigmine and 1 mg atropine were administrated. To rule out intracranial hemorrhages and related eye and optic nerve pathologies, consultation with neurosurgery (in another hospital) and ophthalmology (in the same hospital) services was performed. After extubation, the patient was transferred to the recovery room 2 hours and 20 minutes after the induction of anesthesia, being conscious and oriented with a fairly general state without any complaint of blurred vision. The report of the ophthalmology consultation was as follows: Marcusgunn was negative; the dilated eye was reactive to light, the cornea and the anterior chamber were transparent, the retina vessels had normal appearance, the disc and the capsule were normal without any bleeding, and the lens was normal. Two hours and 10 minutes after ophthalmologic consultation, the patient was transferred to another hospital for neurosurgery consultation. At this point the diameter of the left pupil was 5 mm and the right pupil was 3 mm both being reactive to light. The neurosurgery team performed a brain computed tomography (CT) scan for the patient, the results of which were normal. In the neurosurgery consultation report, it was indicated that the examination of the cranial nerves was normal, the eyes movements were normal, the diameter of the right and the left pupils were respectively 3 mm and 4 mm, and both of them were reactive to light. The patient was returned to the hospital 3 hours and 50 minutes later when the left pupil mydriasis was completely resolved, being in fair general health with stable vital signs. The patient examination was normal days after.

## 3. Discussion

In this case report, a unilateral mydriasis after fixation surgery of right mandibular fracture was presented; however, the mydriasis was completely resolved approximately seven hours after the end of operation without any medical or surgical interventions.

The pupil size is resulted from a balance between sympathetic innervation provided by the trigeminal nerve to the pupillodilator muscle and parasympathetic fibers through the oculomotor nerve innervating sphincter pupillae (5). Although a difference of ≥ 1 mm between the diameter of the pupils is a physiologically observed phenomenon in 20% of the general population (7), an abnormal acute unilateral mydriasisdue to various causes differing from life-threatening etiologies such as intracranial masses and hemorrhage to benign local etiologies, such as eye exposure to mydriatic drugs, is a great concern among physicians. Therefore, properknowledge about eye innervation and the probable causes of abnormal unilateral mydriasis is of great importance for an appropriate evaluation and timely management.

Several studies have reported unilateral mydriasis after surgery, each explaining a unique cause for the event; Schmidt et al. (8) study indicated that eyes exposure to atropine spray was the probable cause for unilateral mydriasis. In another study by Koo et al. (9) it was indicated that the retrograde flow of nasal adrenaline and/or xylometazoline hydrochloride spray, or epinephrine nasal drops through the nasolacrimal duct into the eye and the conjunctival absorption of the drug was another possible explanation. Prielipp (2) reported the patient's unilateral mydriasis as being a result of using phenylephrine/lidocaine spray with a standard oxygen-driven face mask nebulizer. In a study by Burns et al. (10), skin contamination with flea spray containing permethrin and further eye exposure via the child's hand was the probable cause for the child unilateral mydriasis. However, in all of the reported cases, the patients' unilateral mydriasis was resolved after several hours.

In the present case study, brain CT scan and neurosurgery consultation were performed to rule out probable head trauma from the car accident; however, the preoperational brain CT scan showed no evidence of intracranial hemorrhage. Normal postoperative brain CT scan ruled out any life-threatening neurologic causes. Monitored blood pressure and O_2_ saturation stability during the operation decreased the probability of hypoxic trauma as the cause of unilateral mydriasis. Preoperative ophthalmologic examination was normal. Furthermore, the patient's eye was covered during the operation and no nasal spray was used for the patient to indicate that the mydriasis was due to the effect of eye exposure or retrograde flow through the nasolacrimal duct of mydriatic drugs. Injection of anesthetic and haemostatic drugs could not be the probable cause because the injection was performed to the right mandible while mydriasis occurred on the left side. Normal examination of the cranial nerve ruled out previous trauma as the cause of unilateral mydriasis. Moreover, normal preoperational examination ruled out the presence of any congenital disorders. There was no change in the form of the left globe; in addition, postoperative ophthalmologic examination indicated normal condition; therefore, iatrogenic trauma caused by the surgeon during the operation failed to determine the unilateral mydriasis. As a result, there was no explanation for the patient's unilateral mydriasis. The normal examination, the results of the performed consultations and the brain CT scan resolved the surgeon concern about the danger of the event which was resolved after approximately seven hours.

To evaluate a patient with acute unilateral mydriasis, it is suggested to consider life-threatening neurologic causes, in addition to benign causes. Furthermore, knowledge about the anatomy of the eye and innervation and surrounding organs is essential for a timely approach and appropriate management. To differentiate between neurologic and pharmacological causes, it would be beneficial for a pilocarpine test to be immediately performed after the onset of mydriasis; however, the results of the test are not always reliable (11). It is recommended to intermittently evaluate the size of patient’s pupils before, during and after the operation to facilitate the diagnosis of probable causes. Physicians' knowledge about drugs with mydriatic effect can be helpful for rapid diagnosis, decreasing physician concern and reducing patient costs.
